# Medical Image Fusion Based on Fast Finite Shearlet Transform and Sparse Representation

**DOI:** 10.1155/2019/3503267

**Published:** 2019-03-03

**Authors:** Ling Tan, Xin Yu

**Affiliations:** School of Computer and Software, Nanjing University of Information Science & Technology, Nanjing 210044, China

## Abstract

Clinical diagnosis has high requirements for the visual effect of medical images. To obtain rich detail features and clear edges for fusion medical images, an image fusion algorithm FFST-SR-PCNN based on fast finite shearlet transform (FFST) and sparse representation is proposed, aiming at the problem of poor clarity of edge details that is conducive to maintaining the details of source image in current algorithms. Firstly, the source image is decomposed into low-frequency coefficients and high-frequency coefficients by FFST. Secondly, the K-SVD method is used to train the low-frequency coefficients to obtain the overcomplete dictionary *D*, and then the OMP algorithm sparsely encodes the low-frequency coefficients to complete the fusion of the low-frequency coefficients. Then, a high-frequency coefficient is applied to excite a pulse-coupled neural network, and the fusion coefficient of the high-frequency coefficient is selected according to the number of ignitions. Finally, the fused low-frequency coefficient and high-frequency coefficient are reconstructed into the fused medical image by FFST inverse transform. The experimental results show that the image fusion result of the proposed algorithm is about 35% higher than the comparison algorithms for the edge information transfer factor QAB/F index and has achieved good results in both subjective visual effects and objective evaluation indicators.

## 1. Introduction

With the development of imaging devices, different sensors can acquire different information from images of the same scenario [1–4]. In medicine, images of different modes are properly fused to make the source images complementary to each other and thus obtain more informative images [5, 6].

In recent years, the image fusion method based on multiscale geometric analysis has been widely used in the image processing due to its multiresolution characteristics [7]. The wavelet transform [8, 9] is the most typical multiscale analysis method, but it has only three (horizontal, vertical, and diagonal) directions when decomposing an image and thus cannot well represent a two-dimensional image with curve singularity or a high-dimensional function with surface singularity, and it is easy to produce pseudo-Gibbs phenomenon. To solve this problem, multiscale geometric analysis methods such as contourlet transform [10] and shearlet transform [11] have been proposed successively. They have good anisotropy and directional selectivity. Among them, the NSCT is the best one for the image fusion. NSCT has a translation invariance, and it can attenuate the Gibbs effect generated in various types of transformations in the past. But the amount of computational data is too large, the computational complexity is high, and the real-time performance is poor. Compared with NSCT, the shearlet transform [12] fusion algorithm has a more flexible structure, higher computational efficiency, and better fusion effect. However, it uses subsampled in the discretization process, thus it has no translation invariance and is easy to produce pseudo-Gibbs phenomenon near singular points during the image fusion. By cascading the non-subsampling pyramid filter and the shear filter, fast finite shearlet transform (FFST) [13] gets all the advantages of the shearlet transform, avoids the subsampled process, and obtains translation invariance. However, FFST exhibits a problem: the low-frequency coefficients it decomposed are not sparse. Sparse representation (SR) can express the deeper structural characteristics among low-frequency coefficients and make a perfect approximation for the linear combination of a small number of atoms in the dictionary [14]. To extract the fine contour information from the edge of images, highlight the edge features, and get more abundant information, this paper proposed the FFST-SR-PCNN, a medical image fusion algorithm based on the fast finite shearlet transform (FFST) and sparse representation (SR).

## 2. Medical Image Fusion Algorithm Based on FFST-SR-PCNN

**Figure 1:**
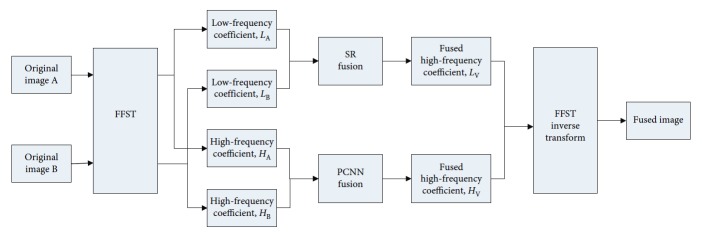
FFST-SR-PCNN medical image fusion algorithm process.

The FFST-SR-PCNN first decomposed the registered source images into low-frequency {*C*_*k*_0__^1^, *C*_*k*_0__^2^} and high-frequency coefficients {*C*_*k*,*l*_^1^, *C*_*k*,*l*_^2^}(*k* > 0, *l* > 0) by FFST, where *k* was the scale of decomposition and *l* was the number of directions of decomposition. Then, low-frequency coefficients were fused by the SR fusion algorithm, and high-frequency coefficients were fused by the fusion algorithm of the simplified PCNN model. Finally, the fused low-frequency and high-frequency coefficients were reconstructed by the inverse FFST to obtain the fused images. The process of FFST-SR-PCNN is illustrated in Figure 1.

### 2.1. Shearlet Transform

Set *A*_*a*_ as the dilation matrix and *S*_*s*_ as the shearlet matrix. They are defined as

(1) $$\begin{array}{c} A_{a}=\left[\begin{array}{cc} a & 0\\ 0 & \sqrt{a} \end{array}\right],\\ S_{s}=\left[\begin{array}{cc} 1 & s\\ 0 & 1 \end{array}\right], \end{array}$$

where *a* ∈ *R* ^+^, *s* ∈ *R*.

For ∀*ψ* ∈ *L* ^2^(*R* ^2^), the shearlet function defined by the dilating, shear, and translation of *ψ* is

(2) $$\begin{array}{c} \psi_{a,s,t}\left(x\right)≔a^{-3/4}\psi \left(A_{a}^{-1}S_{s}^{-1}\left(x-t\right)\right). \end{array}$$

For ∀*f* ∈ *L* ^2^(*R* ^2^), its continuous shearlet transform and corresponding Parseval equation is

(3) $$\begin{array}{c} \text{SH}\left(f\right)\left(a,s,t\right)≔\langle f,\psi_{a,s,t}\rangle =\langle \hat{f},{\hat{\psi }}_{a,s,t}\rangle . \end{array}$$

Specially, define wavelet function *ψ*_1_ and impulse function *ψ*_2_, whose Fourier transform is ${\hat{\psi }}_{1}\left(w\right)$ and ${\hat{\psi }}_{2}\left(w\right)$, respectively.

Set $\hat{\psi }\left(w\right)≔{\hat{\psi }}_{1}\left(w_{1}\right){\hat{\psi }}_{2}\left[w_{1}/w_{2}\right]$; then, $\hat{\psi }\left(w\right)$ fulfills the permissibility. Choose different *w*_1_ and *w*_2_; the frequency domain is separated into different areas, including horizontal cone and vertical cone.

### 2.2. FFST

The shearlet transform generated shearlet functions with different features by scaling, shearing, and translating basis functions. Image decomposition based on the shearlet transform included the following: (1) decompose images into low-frequency and high-frequency subbands at different scales with Laplacian pyramid algorithm; (2) directionally subdivide subbands of different scales with the shear filter to realize multiscale and multidirectional decomposition and to make the size of the decomposed subband images consistent with the source images [15].

To obtain a discrete shearlet transformation, this algorithm discretized the scaling, shearing, and translating parameters in formula (2):

(4) $$\begin{array}{c} a_{j}≔2^{-2j}=\frac{1}{4^{j}},\;j=0,\ldots ,j_{0}-1,\\ s_{j,k}≔k2^{-j},\;-2^{j}\leq k\leq 2^{j},\\ t_{m}≔\frac{m}{N},\;m\in \vartheta , \end{array}$$

where *ϑ*={(*m*_1_, *m*_2_) : *m*_*i*_=0,…, *N* − 1, *i*=1,2} and *j*_0_ represented the scale of decomposition; thus a discrete shearlet was obtained:

(5) $$\begin{array}{c} {\hat{\psi }}_{j,k,m}=\psi \left(A_{a_{j}}^{-1}S_{s,j,k}^{-1}\left(x-t_{m}\right)\right). \end{array}$$

The expression of the frequency domain was

(6) $$\begin{array}{c} {\hat{\psi }}_{j,k,m}\left(\omega \right)=\hat{\psi }\left(A_{a_{j}}^{T}B_{s,j,k}^{T}\omega \right)\exp\left(-2\pi i\langle \omega ,t_{m}\rangle \right)\\ ={\hat{\psi }}_{1}\left(4^{-j}\omega_{1}\right){\hat{\psi }}_{2}\left(2^{j}\frac{\omega_{2}}{\omega_{1}}+k\right)\exp\left(\frac{-2\pi i\langle \omega ,t_{m}\rangle }{N}\right), \end{array}$$

where

(7) $$\begin{array}{c} \Omega ≔\left\{\left(w_{1},w_{2}\right):w_{i}=-\left[\frac{N}{2}\right],\ldots ,\left[\frac{N}{2}\right]-1,\;i=1,2\right\}. \end{array}$$

To obtain the shearlets in the whole frequency domain, |*k*|=2^*j*^ was defined at the intersection of the conical surfaces, and the sum of the shearlets was

(8) $$\begin{array}{c} {\hat{\psi }}_{j,k,m}^{h\times v}≔{\hat{\psi }}_{j,k,m}^{h}+{\hat{\psi }}_{j,k,m}^{v}+{\hat{\psi }}_{j,k,m}^{\times }. \end{array}$$

Thus, the discrete shearlet can be expressed as

(9) $$\begin{array}{c} \text{SH}\left(f\right)\left(j,k,m\right)≔\left\{\begin{array}{cc} \langle f,\phi_{m}\rangle , & \tau =0,\\ \langle f,{\hat{\psi }}_{j,k,m}^{\tau }\rangle , & \tau \in \left\{h,v\right\},\\ \langle f,{\hat{\psi }}_{j,k,m}^{h\times v}\rangle , & \tau =\times ,\left|k\right|=2^{j}, \end{array}\right. \end{array}$$

where *j*=0,…, *j*_0_ − 1, −2^*j*^+1 ≤ 2^*j*^ − 1, *m* ∈ *ϑ*.

The shearlet defined by formula (9) can be realized by a two-dimensional fast Fourier transform algorithm with high computational efficiency. Since FFST has no subsampled process, it owns translation invariance. FFST also has excellent localization characteristics and high directional sensitivity.

### 2.3. Sparse Representation

The basic idea of sparse representation is to represent or approximately represent any signal by the linear combination of a small number of nonzero atoms in a given dictionary [16]. If a signal can be represented or approximated by the linear combination of a small number of atoms in *D* ∈ *R* ^*K*×*N*^, then the mathematical model of sparse representation [14] can be obtained by the following formula:

(10) $$\begin{array}{c} \begin{array}{cc} \underset{A}{\min} & {\left\|A\right\|}_{0},\\ \text{s.t.} & {\left\|X-DA\right\|}_{2}^{2}<\varepsilon , \end{array} \end{array}$$

where dictionary *D*=[*d*_1_, *d*_2_,…, *d*_*N*_] ∈ *R* ^*K*×*N*^ is an overcomplete set; *A* is the coefficient of the sparse representation of signal *X*; ‖*A*‖_0_ is the *L*_0_ norm of *A*; and *ε* is the margin of approximation error.

In FFST-SR-PCNN, first, the K singular value decomposition (K-SVD) method was used to train low-frequency coefficients and obtain the matrix *D* of an overcomplete dictionary. Then, the orthogonal matching pursuit (OMP) optimization algorithm was used to approximate the original signal through the local optimal solution and estimate the coefficient *A* of sparse representation [17]. Finally, the sparse coefficients were fused according to image features adaptively.

With the complete dictionary *D* ∈ *R* ^*K*×*N*^, the objective function equation of the K-SVD algorithm can be written as follows:

(11) $$\begin{array}{c} \begin{array}{cc} \underset{D,\alpha }{\min} & {\left\|X-D\alpha \right\|}_{2}^{2},\\ \text{s.t.} & \forall i,{\left\|\alpha_{i}\right\|}_{0}\leq T_{0}, \end{array} \end{array}$$

where *T*_0_ is the sparse representation of the maximum number of nonzero count in the coefficient, i.e., the maximum sparsity.

Formula (11) is an iterative process. First, suppose the dictionary *D* is fixed, then use the orthogonal matching pursuit (OMP) algorithm to get the sparse matrix; next, fix the matrix and update the dictionary column by column, which means only the first atom in the dictionary is updated.

**Figure 2:**
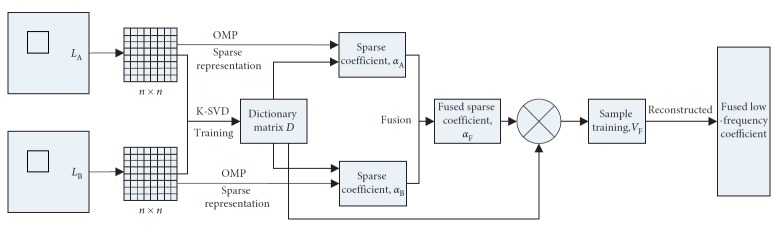
Low-frequency coefficient fusion process based on sparse representation.

The fusion process of low-frequency coefficient based on sparse presentation is illustrated in Figure 2.

In Figure 2, *L*_A_ and *L*_B_ are low-frequency coefficients; *n* × *n* is the size of the sliding window.

### 2.4. Pulse-Coupled Neural Network

Pulse-coupled neural network (PCNN) can combine the input high-frequency coefficients with human visual characteristics to obtain detailed information such as texture, edge, and contour [18]. The mathematical expression of the simplified model is

(12) $$\begin{array}{c} \left\{\begin{array}{c} F_{ij}\left[n\right]=I_{ij},\\ L_{ij}\left[n\right]=\exp\left(-\alpha_{L}\right)L_{ij}\left[n-1\right]+V_{\text{L}}\underset{k,l}{\sum }W_{ijkl}Y_{ij}\left[n-1\right],\\ U_{ij}\left[n\right]=F_{ij}\left[n\right]\left(1+\beta \right)L_{ij}\left[n\right],\\ \theta_{ij}\left[n\right]=\exp\left(-\alpha_{\theta }\right)\theta_{ij}\left[n-1\right]+V_{\theta }Y_{ij}\left[n-1\right],\\ Y_{ij}\left[n-1\right]=\left\{\begin{array}{cc} 1, & U_{ij}\left[n\right]>\theta_{ij}\left[n\right],\\ 0, & U_{ij}\left[n\right]\leq \theta_{ij}\left[n\right], \end{array}\right. \end{array}\right. \end{array}$$

where *n* is the number of iterations; *I*_*ij*_ is the stimulation signal; *Y*_*ij*_ and *U*_*ij*_ are the external input and the internal state, respectively; *F*_*ij*_ is the feedback input; *L*_*ij*_ is the link input; *W*_*ijkl*_ is the connection weight coefficient between neurons; *β*, *θ*_*ij*_, and *α*_*θ*_ are the link strength, the variable threshold input, and the time constant of variable threshold attenuation, respectively; and *V*_L_ and *V*_*θ*_ are amplification coefficients of the link input and the threshold.

High-frequency coefficient fusion used a pixel as the neuronal feedback input to stimulate the simplified PCNN model. SF was

(13) $$\begin{array}{c} F_{ij}={\text{SF}}_{ij}=\sqrt{{\text{RF}}_{ij}^{2}+{\text{CF}}_{ij}^{2}}, \end{array}$$

where the window size was 3 × 3; RF_*ij*_ and CF_*ij*_ were

(14) $$\begin{array}{c} {\text{RF}}_{ij}=\sqrt{\frac{1}{M\times N}\overset{M}{\underset{i=1}{\sum }}\overset{N}{\underset{j=2}{\sum }}{\left[X\left(i,j\right)-X\left(i,j-1\right)\right]}^{2}},\\ {\text{CF}}_{ij}=\sqrt{\frac{1}{M\times N}\overset{M}{\underset{i=2}{\sum }}\overset{N}{\underset{j=1}{\sum }}{\left[X\left(i,j\right)-X\left(i-1,j\right)\right]}^{2}}. \end{array}$$

It got ignition maps through PCNN ignition and selected fusion coefficients according to the number of ignition times.

## 3. Implementation of FFST-SR-PCNN

### 3.1. Rules of Low-Frequency Coefficient Fusion

The process was implemented as follows:

 
*Step 1*. Decompose the source images A and B with the registered size *M* × *N* by FFST to obtain the low-frequency coefficient and the high-frequency coefficient.
 
*Step 2*. Using a sliding window with a step size of one pixel *S* and a size *n* × *n*, the low-frequency coefficients *L*_A_ and *L*_B_ are subjected to block processing to obtain (*N*+*n* − 1) × (*M*+*n* − 1) image subblocks, and the image subblocks are converted into column vectors to obtain a sample training matrix *V*_A_ and *V*_B_.
 
*Step 3*. Do iterative operation for sample matrix with K-SVD and obtain overcomplete dictionary matrix *D* of low-frequency efficient.
 
*Step 4*. Estimate the sparse coefficient of *V*_A_ and *V*_B_ with OMP algorithm and obtain sparse coefficient matrix *α*_A_ and *α*_B_. The *i*th column sparse coefficient matrix will be fused as follows.
  *Case 1*. If the *L*_1_ norm of *α*_A_ is larger than *L*_1_ norm of *α*_B_, then fuse with equation (15):

(15) $$\begin{array}{c} \alpha_{\text{F}}^{i}=\left\{\begin{array}{cc} \alpha_{\text{A}}^{i}+\frac{1}{2}\alpha_{\text{B}}^{i}, & \text{if}\;\;\alpha_{\text{A}}^{i}<\alpha_{\text{B}}^{i},\alpha_{\text{A}}^{i}\cdot \alpha_{\text{B}}^{i}<0,\\ \alpha_{\text{A}}^{i}, & \text{otherwise}. \end{array}\right. \end{array}$$

 
*Case 2*. If the *L*_1_ norm of *α*_A_ is smaller than *L*_1_ norm of *α*_B_, then fuse with equation (16):

(16) $$\begin{array}{c} \alpha_{\text{F}}^{i}=\left\{\begin{array}{cc} \alpha_{\text{B}}^{i}+\frac{1}{2}\alpha_{\text{A}}^{i}, & \text{if}\;\;\alpha_{\text{A}}^{i}>\alpha_{\text{B}}^{i},\alpha_{\text{A}}^{i}\cdot \alpha_{\text{B}}^{i}<0,\\ \alpha_{\text{B}}^{i}, & \text{otherwise}. \end{array}\right. \end{array}$$

 
*Case 3*. If the *L*_1_ norm of *α*_A_ is equal to *L*_1_ norm of *α*_B_, then fuse with equation (17):

(17) $$\begin{array}{c} \alpha_{\text{F}}^{i}=\left\{\begin{array}{cc} \alpha_{\text{A}}^{i}+\frac{1}{2}\alpha_{\text{B}}^{i}, & \text{if}\;\;\alpha_{\text{A}}^{i}>\alpha_{\text{B}}^{i},\alpha_{\text{A}}^{i}\cdot \alpha_{\text{B}}^{i}<0,\\ \alpha_{\text{B}}^{i}+\frac{1}{2}\alpha_{\text{A}}^{i}, & \text{if}\;\;\alpha_{\text{A}}^{i}<\alpha_{\text{B}}^{i},\alpha_{\text{A}}^{i}\cdot \alpha_{\text{B}}^{i}<0,\\ \frac{\alpha_{\text{A}}^{i}+\alpha_{\text{B}}^{i}}{2}, & \text{otherwise}, \end{array}\right. \end{array}$$

  where *α*_A_^*i*^ and *α*_B_^*i*^ are the *i*th column sparse coefficient matrix of *α*_A_ and *α*_B_, respectively; *α*_F_^*i*^ is the *i*th column fused sparse coefficient matrix.
 
*Step 5*. Multiply overcomplete dictionary matrix *D* and fused sparse coefficient matrix *α*_F_. Fused sample training matrix *V*_F_ is

(18) $$\begin{array}{c} V_{\text{F}}=D\alpha_{\text{F}}. \end{array}$$

 
*Step 6*. Turn the columns of *V*_F_ into data subblocks, reconstruct data subblocks, and obtain low-frequency fusion coefficient.

### 3.2. Rules of High-Frequency Coefficient Fusion

The process was implemented as follows:

 
*Step 1*. Calculate the neighborhood spatial frequency SF_A_ and SF_B_ of the high-frequency coefficients *H*_A_ and *H*_B_ according to equation (13) and use it as the link strength values of the neurons.
 
*Step 2*. Initialization: *L*_*ij*_(0)=*U*_*ij*_(0)=*θ*_*ij*_(0)=0. Now neurons are in off state, i.e., *Y*_*ij*_(0)=0 the resulting pulse is *O*_*ij*_(0)=0.
 
*Step 3*. Compute *L*_*ij*_[*n*], *U*_*ij*_[*n*], *θ*_*ij*_[*n*] and *Y*_*ij*_[*n*] according to equation (12).
 
*Step 4*. Compare the output threshold (ignition frequency) of firing time at the pixels of fire mapping image *O*_A_, *O*_B_; the high-frequency fused coefficient *H*_F_(*i*, *j*) is

(19) $$\begin{array}{c} H_{\text{F}}\left(i,j\right)=\left\{\begin{array}{cc} H_{\text{A}}\left(i,j\right), & \text{if}\;\;O_{\text{A}}\left(i,j\right)>O_{\text{B}}\left(i,j\right),\\ H_{\text{B}}\left(i,j\right), & \text{if}\;\;O_{\text{A}}\left(i,j\right)<O_{\text{B}}\left(i,j\right),\\ \frac{H_{\text{A}}\left(i,j\right)+H_{\text{B}}\left(i,j\right)}{2}, & \text{if}\;\;O_{\text{A}}\left(i,j\right)=O_{\text{B}}\left(i,j\right). \end{array}\right. \end{array}$$

## 4. Experimental Results and Analysis

In order to verify the effectiveness of FFST-SR-PCNN, five representative algorithms were selected as the controls for medical image fusion experiments. Five indicators including spatial frequency (SF), average gradient (AG), mutual information (MI), edge information transfer factor QAB/F (high-weight evaluation indicator) [19–22], and running time (RT) were used to make objective evaluation. Comparing algorithm 1 was a fusion algorithm proposed in [23] for images based on PCNN. Comparing algorithm 2 was an improved fusion algorithm proposed in [24] for medical images based on NSCT and adaptive PCNN. Comparing algorithm 3 was a fusion algorithm proposed in [25] for medical images based on SR and neural network. Comparing algorithm 4 was a fusion algorithm proposed in [26] for multimode medical images based on NSCT and Log-Gabor energy. Comparing algorithm 5 was a fusion algorithm proposed in [27] for medical images based on non-subsampled Shearlet transform and parameter adaptive pulse-coupled neural network.

### 4.1. Gray Image Fusion Experiment

**Figure 3:**
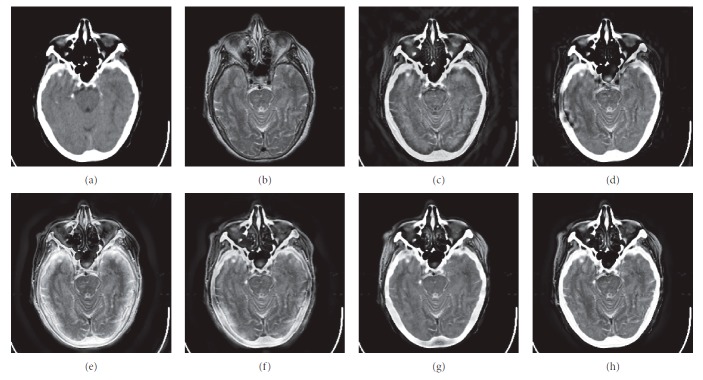
CT/MR-T2 medical image fusion results. (a) CT original image. (b) MR-T2 original image. (c) Method 1. (d) Method 2. (e) Method 3. (f) Method 4. (g) Method 5. (h) FFST-SR-PCNN.

**Figure 4 fig4:**
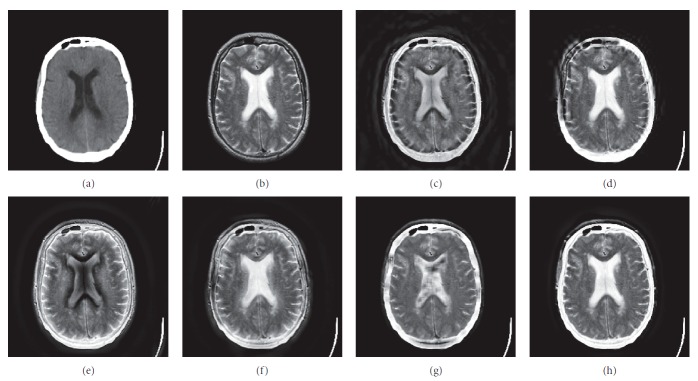
CT/MR-T2 medical image fusion results. (a) CT original image. (b) MR-T2 original image. (c) Method 1. (d) Method 2. (e) Method 3. (f) Method 4. (g) Method 5. (h) FFST-SR-PCNN.

**Figure 5 fig5:**
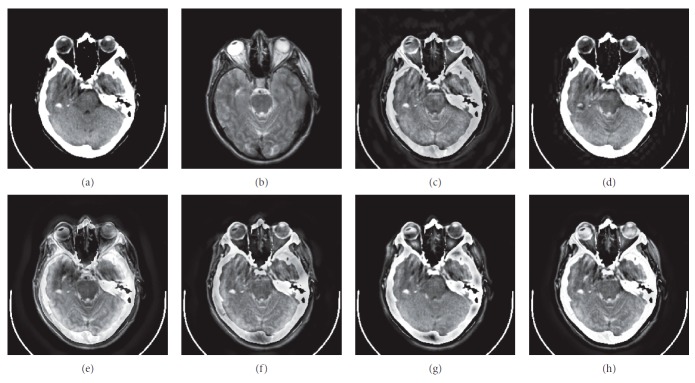
CT/MR-T2 medical image fusion results. (a) CT original image. (b) MR-T2 original image. (c) Method 1. (d) Method 2. (e) Method 3. (f) Method 4. (g) Method 5. (h) FFST-SR-PCNN.

**Figure 6 fig6:**
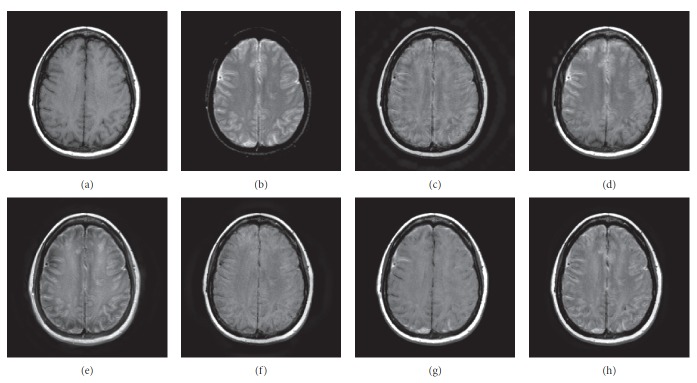
MR-T1/MR-T2 medical image fusion results. (a) MR-T1 original image. (b) MR-T2 original image. (c) Method 1. (d) Method 2. (e) Method 3. (f) Method 4. (g) Method 5. (h) FFST-SR-PCNN.

**Figure 7 fig7:**
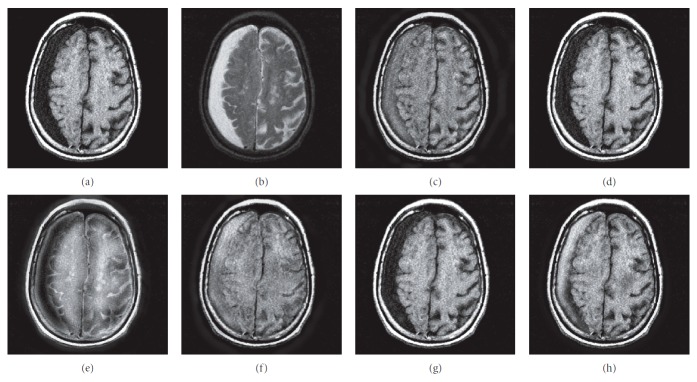
MR-T1/MR-T2 medical image fusion results. (a) MR-T1 original image. (b) MR-T2 original image. (c) Method 1. (d) Method 2. (e) Method 3. (f) Method 4. (g) Method 5. (h) FFST-SR-PCNN.

**Figure 8:**
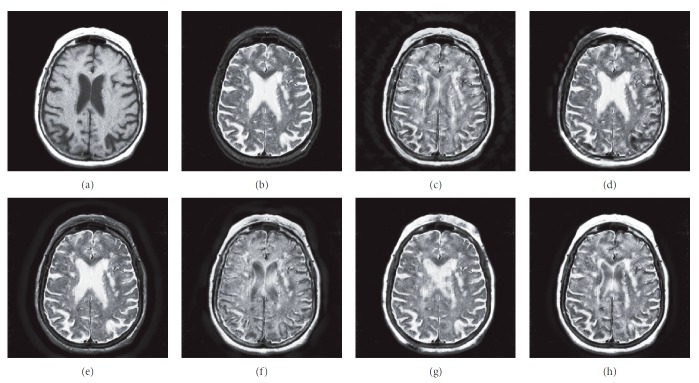
MR-T1/MR-T2 medical image fusion results. (a) MR-T1 original image. (b) MR-T2 original image. (c) Method 1. (d) Method 2. (e) Method 3. (f) Method 4. (g) Method 5. (h) FFST-SR-PCNN.

In this experiment, six pairs of brain images in different states were selected for fusion. The first three pairs are CT/MR-T2 images and the last three pairs are MR-T1/MR-T2 images. The resulting images fused by different algorithms are shown in Figures 3 [Fig fig4] [Fig fig5] [Fig fig6] [Fig fig7]–8, and their objective quality evaluation indicators are listed in Tables 1 [Table tab2] [Table tab3] [Table tab4] [Table tab5]–6.

According to Figures 3 [Fig fig4] [Fig fig5] [Fig fig6] [Fig fig7]–8, comparing algorithm 1 gave poor performance compared to the source images in the presentation of detailed feature information and had horizontal and vertical blocking effects (Figures 3(c), 4(c), 5(c), 6(c), 7(c), and 8(c)). Comparing algorithm 2 gave poor performance compared to the source MR-T2 image in the presentation of detailed edge information and had blurry edge details (Figures 3(d), 4(d), 5(d), 6(d), 7(d), and 8(d)). Comparing algorithm 3 had low overall contrast and blurred edge details (Figures 3(e), 4(e), 5(e), 6(e), 7(e), and 8(e)). Comparing algorithm 4 had blurry edge details (Figures 3(f), 4(f), 5(f), 6(f), 7(f), and 8(f)). Comparing algorithm 5 had low contrast in the upper right corner (Figures 3(g), 4(g), 5(g), 6(g), 7(g), and 8(g)). FFST-SR-PCNN fully retained the feature information of the source images, without dark lines and low contrast (Figures 3(h), 4(h), 5(h), 6(h), 7(h), and 8(h)). From the evaluation indicators in Tables 1 [Table tab2] [Table tab3] [Table tab4] [Table tab5]–6, FFST-SR-PCNN had better performance than the other five comparing algorithms on QAB/F by an average increase of 15.5%. FFST-SR-PCNN is not always the best one in each individual evaluation indicators, but it never ranked less than top three. It can be seen that the computational efficiency of FFST-SR-PCNN was lower than comparing algorithm 5 (average 34.8% lower), while higher than the other four methods (average 34.6%, 65%, 63.7%, and 48.5% higher, respectively). This is because the number of iterations of the comparison algorithm 5 is relatively small, but its other indicators were not as good as FFST-SR-PCNN. Totally, FFST-SR-PCNN had the best effect and can provide better fused medical images with relative lower computing cost.

### 4.2. Color Image Fusion Experiment

**Figure 9:**
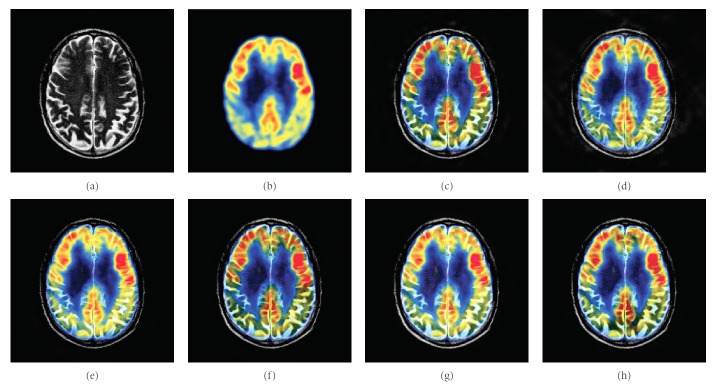
MR-T2/PET medical image fusion results. (a) MR-T2 original image. (b) PET original image. (c) Method 1. (d) Method 2. (e) Method 3. (f) Method 4. (g) Method 5. (h) FFST-SR-PCNN.

**Figure 10 fig10:**
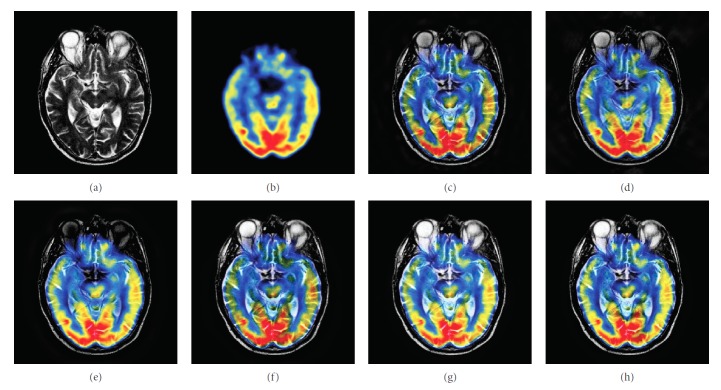
MR-T2/PET medical image fusion results. (a) MR-T2 original image. (b) PET original image. (c) Method 1. (d) Method 2. (e) Method 3. (f) Method 4. (g) Method 5. (h) FFST-SR-PCNN.

**Figure 11 fig11:**
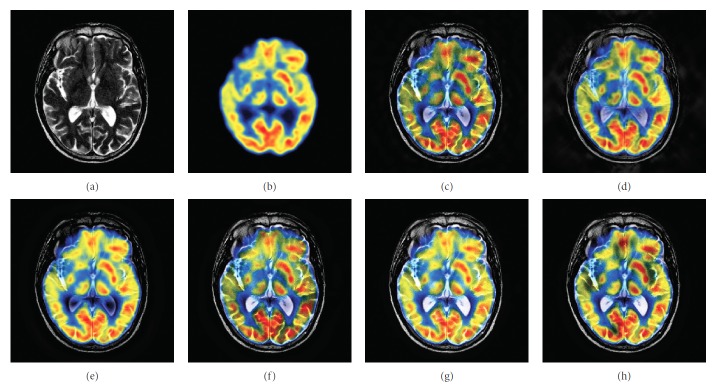
MR-T2/PET medical image fusion results. (a) MR-T2 original image. (b) PET original image. (c) Method 1. (d) Method 2. (e) Method 3. (f) Method 4. (g) Method 5. (h) FFST-SR-PCNN.

**Figure 12 fig12:**
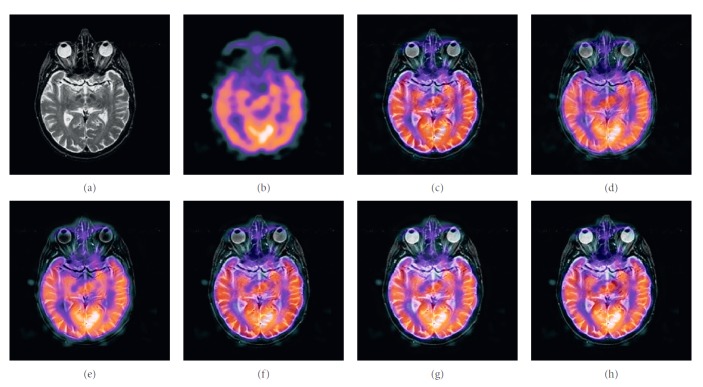
MR-T2/SPECT medical image fusion results. (a) MR-T2 original image. (b) SPECT original image. (c) Method 1. (d) Method 2. (e) Method 3. (f) Method 4. (g) Method 5. (h) FFST-SR-PCNN.

**Figure 13 fig13:**
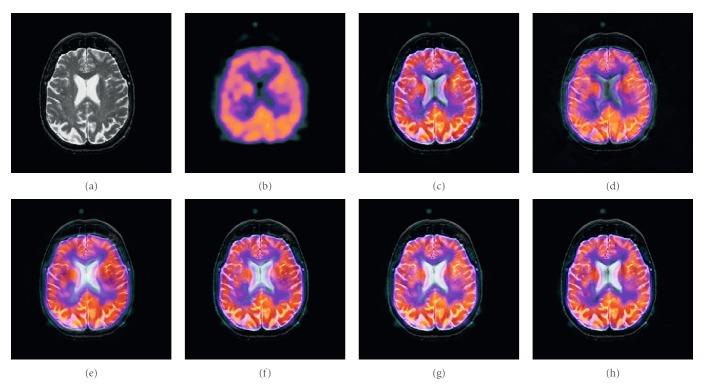
MR-T2/SPECT medical image fusion results. (a) MR-T2 original image. (b) SPECT original image. (c) Method 1. (d) Method 2. (e) Method 3. (f) Method 4. (g) Method 5. (h) FFST-SR-PCNN.

**Figure 14:**
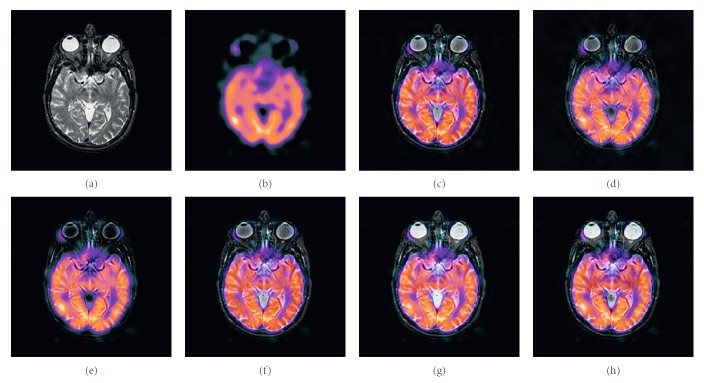
MR-T2/SPECT medical image fusion results. (a) MR-T2 original image. (b) SPECT original image. (c) Method 1. (d) Method 2. (e) Method 3. (f) Method 4. (g) Method 5. (h) FFST-SR-PCNN.

In this experiment, six pairs of brain images in different states were selected for fusion. The first three pairs are MR-T2/PET images and the last three pairs are MR-T2/SPECT images. The resulting images fused by different algorithms are shown in Figures 9 [Fig fig10] [Fig fig11] [Fig fig12] [Fig fig13]–14, and their objective quality evaluation indicators are listed in Tables 7 [Table tab8] [Table tab9] [Table tab10] [Table tab11]–12.

According to Figures 9 [Fig fig10] [Fig fig11] [Fig fig12] [Fig fig13]–14, comparing algorithm 1 gave poor performance compared to the source image in the presentation of detailed feature information and had widespread blocking effects (Figures 9(c), 10(c), 11(c), 12(c), 13(c), and 14(c)). Comparing algorithm 2 retained most feature information from the source images, but the fused image had low overall contrast (Figures 9(d), 10(d), 11(d), 12(d), 13(d), and 14(d)). Comparing algorithm 3 had blurred edge contours compared to the source image (Figures 9(e), 10(e), 11(e), 12(e), 13(e), and 14(e)). Comparing algorithm 4 retained most of the feature information from the source image, but the edge contours are blurred (Figures 9(f), 10(f), 11(f), 12(f), 13(f), and 14(f)). Comparing algorithm 5 has clearer details than the other four algorithms (method 1 to method 4), but its contrast is still somewhat low (Figures 9(g), 10(g), 11(g), 12(g), 13(g), and 14(g)). The FFST-SR-PCNN method fully retained the feature information from the source images, without low contrast and blocking effects (Figures 9(h), 10(h), 11(h), 12(h), 13(h), and 14(h)). From the evaluation indicators in Tables 7 [Table tab8] [Table tab9] [Table tab10] [Table tab11]–12, FFST-SR-PCNN had better performance than the other five comparing algorithms on QAB/F by an average increase of 31.7%. FFST-SR-PCNN is not always the best one in each individual evaluation indicators, but it never ranked less than top two. It can be seen that the computational efficiency of the proposed method was lower than comparing algorithm 5 (average 17.7% lower), while higher than the other four methods (average 40.35%, 76.8%, 69.8%, and 64.4% higher, respectively). This is because the number of iterations of the comparison algorithm 5 is relatively small, but its other indicators were not as good as the proposed algorithm. Overall, FFST-SR-PCNN had the best effect and can provide better fused medical images with relative lower computing cost.

Taken above gray images and color images fusion results together, FFST-SR-PCNN can achieve better fusion performance in edge sharpness, change intensity, and contrast.

## 5. Conclusion

To promote the fusion performance of unimodal medical images, this thesis proposed a FFST-SR-PCNN algorithm based on FFST, sparse presentation, and pulse-coupled neural network. It has excellent detail delineation and can efficiently extract the feature information of images, thus enhanced the overall performance of the fusion results. The performance of FFST-SR-PCNN is evaluated by several experiments. In the comparing experiments with 5 comparison algorithms, all single-evaluation indexes of our algorithm are ranked in the top three; the comprehensive evaluation index of our algorithm has best result, and its QAB/F is higher than other 5 comparison algorithms. In terms of subjective manner, FFST-SR-PCNN can efficiently express the marginal information of images and make the details of fusion image clearer, with more smooth edges. Thus, it has better subjective visual effects.

## Figures and Tables

**Table 1:** Quality assessment of CT/MR-T2 medical image fusion.

Index	Method 1	Method 2	Method 3	Method 4	Method 5	FFST-SR-PCNN
SF	34.2861	34.3181	30.7780	30.6366	36.6748	**36.9338**
AG	9.6513	8.2010	**10.0870**	9.6272	9.2317	9.6804
MI	2.1254	2.2705	2.0769	2.2199	**2.9953**	2.5248
QAB/F	0.5190	0.4850	0.4939	0.5257	0.5843	**0.5995**
RT/s	16.2069	32.4554	30.5628	22.5256	**8.7295**	11.3624

**Table 2 tab2:** Quality assessment of CT/MR-T2 medical image fusion.

Index	Method 1	Method 2	Method 3	Method 4	Method 5	FFST-SR-PCNN
SF	27.0626	26.9760	26.3291	23.9678	28.7623	**29.3344**
AG	7.5929	6.7873	**7.9253**	7.2387	6.7479	7.2640
MI	2.1941	2.6101	2.2457	2.3168	2.9609	**3.0805**
QAB/F	0.4617	0.4088	0.5313	0.4733	0.5161	**0.5473**
RT/s	16.2266	32.3894	29.9843	22.0427	**7.9451**	10.1755

**Table 3 tab3:** Quality assessment of CT/MR-T2 medical image fusion.

Index	Method 1	Method 2	Method 3	Method 4	Method 5	FFST-SR-PCNN
SF	37.5717	41.0988	36.5295	38.8050	40.0197	**41.7215**
AG	9.8877	8.9200	10.0862	10.2808	10.4221	**10.4347**
MI	2.0886	2.3744	2.0724	2.1990	2.4176	**2.4719**
QAB/F	0.5559	0.5582	0.5242	0.6148	0.6300	**0.6516**
RT/s	16.2690	31.5377	30.1011	22.4004	**8.3358**	11.7004

**Table 4 tab4:** Quality assessment of MR-T1/MR-T2 medical image fusion.

Index	Method 1	Method 2	Method 3	Method 4	Method 5	FFST-SR-PCNN
SF	22.7160	22.6900	21.5347	22.7164	24.3787	**24.4962**
AG	6.4857	6.3629	6.6983	6.4737	6.5910	**6.8862**
MI	2.3686	2.6473	2.4908	2.4517	**2.9919**	2.7319
QAB/F	0.5204	0.5614	0.6105	0.5686	0.6261	**0.6416**
RT/s	15.3972	27.2017	29.6786	22.8392	**7.8440**	8.5966

**Table 5 tab5:** Quality assessment of MR-T1/MR-T2 medical image fusion.

Index	Method 1	Method 2	Method 3	Method 4	Method 5	FFST-SR-PCNN
SF	33.0368	**35.8930**	26.6605	30.3170	33.7416	34.2538
AG	12.8183	**13.6667**	10.2990	11.7227	12.8446	13.3015
MI	2.7059	**4.0357**	2.4716	2.6096	2.9629	3.2689
QAB/F	0.6086	**0.6540**	0.4126	0.5333	0.5285	0.6353
RT/s	16.2790	31.6667	31.1026	22.0803	**8.0882**	12.4248

**Table 6:** Quality assessment of MR-T1/MR-T2 medical image fusion.

Index	Method 1	Method 2	Method 3	Method 4	Method 5	FFST-SR-PCNN
SF	25.6557	25.1765	23.7800	23.2217	26.4393	**27.8123**
AG	9.8227	9.3917	8.9306	8.9716	9.5509	**10.2213**
MI	2.5279	3.1267	3.1352	2.5935	**3.7742**	3.1781
QAB/F	0.4643	0.4707	0.5005	0.4656	0.5478	**0.5724**
RT/s	16.2837	34.0250	31.1384	22.7084	**8.2634**	12.1475

**Table 7:** Quality assessment of MR-T2/PET medical image fusion.

Index	Method 1	Method 2	Method 3	Method 4	Method 5	FFST-SR-PCNN
SF	27.9886	24.4599	24.5435	28.1239	28.1658	**28.7088**
AG	8.6039	7.9211	7.0367	8.6999	8.5409	**8.9234**
MI	3.1791	3.0843	3.2350	3.2391	3.3424	**3.7138**
QAB/F	0.5920	0.4431	0.4673	0.6186	0.5797	**0.6882**
RT/s	15.5014	39.0562	31.0281	26.6560	**9.1450**	9.3991

**Table 8 tab8:** Quality assessment of MR-T2/PET medical image fusion.

Index	Method 1	Method 2	Method 3	Method 4	Method 5	FFST-SR-PCNN
SF	32.6319	27.9935	28.0245	33.7764	34.2547	**34.5131**
AG	10.9999	9.4439	8.8861	11.5490	**11.8755**	11.5588
MI	3.3142	3.2200	3.2471	3.4545	3.7286	**4.0487**
QAB/F	0.5886	0.4417	0.4566	0.6541	0.6331	**0.7145**
RT/s	15.4448	39.8451	30.4667	25.3966	**7.7208**	9.9195

**Table 9 tab9:** Quality assessment of MR-T2/PET medical image fusion.

Index	Method 1	Method 2	Method 3	Method 4	Method 5	FFST-SR-PCNN
SF	32.1710	26.2209	26.6609	32.8288	33.5603	**34.0538**
AG	11.0293	9.3185	8.5356	11.3710	11.4366	**11.6015**
MI	3.3244	3.2592	3.3420	3.4270	3.6002	**3.9820**
QAB/F	0.5580	0.4026	0.4468	0.6060	0.5819	**0.6898**
RT/s	15.4377	40.0521	30.5628	26.3830	**7.7023**	10.0894

**Table 10 tab10:** Quality assessment of MR-T2/SPECT medical image fusion.

Index	Method 1	Method 2	Method 3	Method 4	Method 5	FFST-SR-PCNN
SF	22.1254	17.6301	17.4476	21.6041	**22.2932**	21.7161
AG	**7.4238**	6.2468	5.9028	7.1767	7.4044	7.0624
MI	2.7010	2.6122	2.7375	2.7932	2.9168	**3.8262**
QAB/F	0.6647	0.3967	0.4266	0.6481	0.6849	**0.7154**
RT/s	15.4280	40.6601	30.8525	26.2191	**7.4911**	8.9699

**Table 11 tab11:** Quality assessment of MR-T2/SPECT medical image fusion.

Index	Method 1	Method 2	Method 3	Method 4	Method 5	FFST-SR-PCNN
SF	**19.0298**	15.1207	15.2854	18.7206	19.0044	18.3672
AG	**5.8012**	5.1296	4.8812	5.5770	5.7915	5.3554
MI	2.5702	2.3812	2.5202	2.6742	2.7730	**3.4318**
QAB/F	0.6692	0.3700	0.4581	0.6589	0.6668	**0.6773**
RT/s	15.4179	38.9686	30.0630	26.3533	**7.5471**	8.3015

**Table 12:** Quality assessment of MR-T2/SPECT medical image fusion.

Index	Method 1	Method 2	Method 3	Method 4	Method 5	FFST-SR-PCNN
SF	22.0008	18.5584	17.6392	21.7631	**22.2414**	21.5873
AG	**7.1240**	6.3536	5.5604	6.9306	7.1145	6.9346
MI	22.4242	2.2906	2.3527	2.5019	2.6839	**3.5690**
QAB/F	0.6753	0.4438	0.4188	0.6873	0.6915	**0.7207**
RT/s	15.4405	39.9952	30.1950	25.8698	**7.5521**	8.6035

## Data Availability

The data used to support the findings of this study are included within the article.
